# Cooperative Analysis of Structural Dynamics in RNA-Protein Complexes by Single-Molecule Förster Resonance Energy Transfer Spectroscopy

**DOI:** 10.3390/molecules25092057

**Published:** 2020-04-28

**Authors:** Nathalie Meiser, Christin Fuks, Martin Hengesbach

**Affiliations:** Institute for Organic Chemistry and Chemical Biology, Goethe-University Frankfurt, Max-von-Laue-Str. 7, 60438 Frankfurt, Germany

**Keywords:** RNA-protein complex (RNP), single-molecule förster resonance energy transfer (smFRET) spectroscopy, RNP dynamics, spliceosome, G quadruplex helicase RHAU, telomerase

## Abstract

RNA-protein complexes (RNPs) are essential components in a variety of cellular processes, and oftentimes exhibit complex structures and show mechanisms that are highly dynamic in conformation and structure. However, biochemical and structural biology approaches are mostly not able to fully elucidate the structurally and especially conformationally dynamic and heterogeneous nature of these RNPs, to which end single molecule Förster resonance energy transfer (smFRET) spectroscopy can be harnessed to fill this gap. Here we summarize the advantages of strategic smFRET studies to investigate RNP dynamics, complemented by structural and biochemical data. Focusing on recent smFRET studies of three essential biological systems, we demonstrate that investigation of RNPs on a single molecule level can answer important functional questions that remained elusive with structural or biochemical approaches alone: The complex structural rearrangements throughout the splicing cycle, unwinding dynamics of the G-quadruplex (G4) helicase RHAU, and aspects in telomere maintenance regulation and synthesis.

## 1. Introduction

Many cellular processes rely on the faithful interaction between proteins and RNA, which constitute most major molecular machineries. For some of these RNA-protein complexes (RNPs), structural data has been available for quite some time, the most prominent example being the ribosome [1]. However, the underlying molecular processes are highly complex and oftentimes exhibit an unexpected degree of conformational dynamics, rendering their analysis technically demanding. In addition, assessing molecular or conformational dynamic heterogeneity is as difficult as acquiring more exhaustive descriptions of such processes. For the latter, one current trend is to employ integrative structural biology approaches, combining analytical toolsets with structural and biochemical data to provide more detailed characterization of such processes. Such ensemble techniques are however limited when it comes to the description of heterogeneous conformations and dynamics; in these cases, a number of methods that allow for the dissection of such heterogeneous populations are required. Here, techniques that provide analytics on the level of individual molecules have proven to be of great value, as they allow the assessment of both heterogeneity and conformational dynamics at the same time. The most prominent method that has been used for quite a number of different biological systems is single molecule FRET spectroscopy.

FRET is based on the non-radiative energy transfer from a donor to an acceptor fluorophore that show a spectral overlap of emission and absorption spectra, respectively. This transfer is distance dependent to the sixth power and scales with the FRET pair specific Förster radius (R_0_), leading to high FRET efficiencies when the two fluorophores come into closer proximity. This allows applying FRET spectroscopy as a molecular ruler, covering distances ranging from approximately 2 to 10 nm. First FRET experiments were performed in an ensemble approach, therefore averaging over all molecules in potentially different conformations. However, applications in a single molecule setup allow observation of individual, non-synchronized molecules with different conformations and dynamics. Detection in single molecule resolution is achieved by applying total internal reflection fluorescence (TIRF) microscopy or confocal based setups. However, TIRF based approaches are the most widely used weapon of choice. This technique requires immobilization of the FRET-labeled constructs to the imaged surface. Independent of the imaging technique, strategic construct design is a crucial step to achieve significant results using smFRET. Labeling sites are chosen based on structural data (if available) corresponding to an expected high distance variance upon conformational changes preferably crossing the Förster radius. In studies on RNP dynamics, RNA or proteins can be targeted for fluorophore labeling [2,3,4,5]. The experimentally generated FRET trajectories then provide information on the conformational distribution in equilibrium, the time-resolved interconversion between different states, and can identify intermediates and folding pathways.

Within the last years, a wide array of approaches for various systems has been developed; ranging from ultrashort dynamics of unfolded proteins [6] over the analysis of individual RNAs to highly complex systems like helicases [7,8], the ribosome [9,10,11], argonaute proteins [12,13], or polymerases [5,14]. 

Here, we want to showcase selected recent examples on three RNPs that have been presented within the last years and immensely benefited from smFRET approaches: the spliceosome [15,16,17,18], a G-quadruplex resolving helicase [19,20,21,22], and telomere maintaining complexes [23,24,25]. All of these have in common that the insights from single molecule FRET experiments are complemented by structural and biochemical analysis, and we will emphasize how these approaches are mutually beneficial.

## 2. Spliceosome

Splicing is an essential process that allows eukaryotic cells to derive different protein coding mRNAs from the same genomic sequence. This is achieved by cutting and rejoining the pre-mRNA in a site-specific fashion. The RNP complex that catalyzes this highly regulated process is called spliceosome, which is in itself highly dynamic in its structure as well as its molecular composition; among the components are five unique spliceosomal RNAs (U1, U2, U4–6) and a varying number (~100) of proteins. The recent advances in structural biology have yielded the first models of the spliceosome core structure [26,27,28], aided by novel approaches that facilitated purification of spliceosomes at defined steps of the splicing cycle. In retrospect, the highly conformationally and compositionally dynamic nature of the splicing process was one of the major obstacles in many of the approaches that aimed at investigating the structure on the molecular level.

Several crucial steps can be defined that are required for efficient splicing: (1) Recognition of the 5′ splice site (5′SS), which occurs by binding of the pre-mRNA to the U1 RNA (commitment complex 2), (2) Binding of U2 RNA to the branch site (A-complex), (3) Recruitment of the trimeric snRNP (tri-snRNP) into the complex (B-complex), (4) multiple conformational rearrangement and remodeling steps, during which U1 and U4 are removed from the complex (B^act^-complex), (5) formation of the catalytic core by U2 and U6, (6) transesterification and formation of a lariat structure (C-complex), (7) additional remodeling steps, and (8) second transesterification and release of the ligated exons.

While this list is neither exhaustive nor sufficiently detailed to fully describe the splicing process, it provides an impression of the complexity of the splicing cycle. It also illustrates the need for techniques that faithfully report on dynamics within each of these steps.

Recent work in several labs was focusing on different steps throughout the splicing cycle: During spliceosome assembly, the role of Prp5 on U2 and pre-mRNA was investigated [18,29]. For conformational rearrangements during the transesterification reaction, dynamics of U2 RNA and the effect of nucleotide modifications, magnesium ion binding, and the interaction with Cus2 were taken into focus [16,17]. To generate a more holistic picture of the splicing cycle, an unbiased classification method for structural dynamics was established [15].

## 3. The Role of Prp5 on Spliceosome Assembly

**Figure 1:**
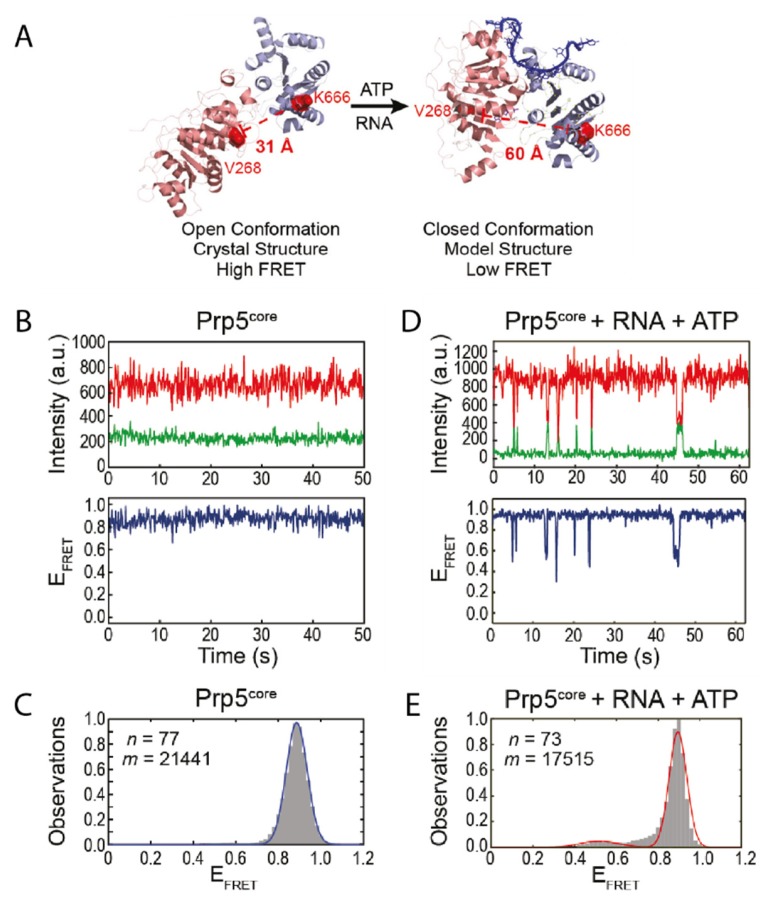
Conformational changes within Prp5. (**A**) Crystal structure of Prp5 in the twisted open conformation and modelled structure of closed conformation. The two RecA-like domains of Prp5 are shown in pink and purple and the RNA in the closed conformation in blue. The residues mutated for fluorophore introduction are indicated in red. (**B**) Exemplary Förster resonance energy transfer (FRET) trajectory of Prp5 alone and (**C**) the corresponding FRET histogram fitted with one peak (blue line). (**D**) FRET behavior of Prp5 in the presence of poly (A) RNA and ATP and (**E**) the corresponding FRET histogram fitted with two peaks (red line). This Figure was taken from Beier et al. [18] and is used under Creative Commons Non-Commercial License (CC-BY NC).

The DEAD-box ATPase Prp5 was found to be essential for initiating the splicing process [30]. It mediates recognition of the pre-mRNA branch site by the spliceosomal RNA U2 while undergoing a conformational switch [31]. This step provides proofreading functionality for the branch site recognition [32,33], while it is no longer needed in later steps of the splicing process [32]. The structure of Prp5 has been solved to 1.95 Å showing that it assumes a twisted open conformation (Figure 1A) [31]. To understand the mode of action of the protein it is inevitable to not only consider snapshots, but also to identify possible short-lived conformations and how they are influenced by certain factors. For Prp5 specifically this might be an additional, closed conformation as observed for other DEAD-box helicases [7,34].

Based on the available structural information, Beier et al. designed a smFRET construct using cysteine mutations of Prp5 [18]. Within this construct, the twisted open conformation would lead to a small distance between the FRET pair, resulting in high FRET efficiency (Figure 1A). The assumed conformational change however would increase the distance, leading to a lower FRET efficiency in the closed conformation. In the absence of any cofactor or substrate, the protein assumes the open conformation as expected (Figure 1B,C); this is also in line with studies on other DEAD-box helicases [7,34]. Only when adding both ATP and substrate however, the construct underwent transient, short-lived transitions into the closed conformation (Figure 1D,E). The authors showed that their smFRET results can be coupled with other biochemical methods to gain an even more detailed understanding of the system. A biochemical ATPase assay confirmed that ATP hydrolysis was increased in presence of RNA. The conformational switch was also disfavored in presence of ADP, but the lifetime of the closed state was increased in presence of the nonhydrolyzable ADPNP. Mutations of Prp5 were introduced that either increased or decreased specificity of the branch site recognition. Conformational analysis of these mutants showed that there is a strong correlation between the structural dynamics of Prp5 mutants, i.e., the lifetime of their closed and open state, their ATP hydrolysis activity, and their impact on branch site recognition [18].

## 4. U2 Toggling

**Figure 2:**
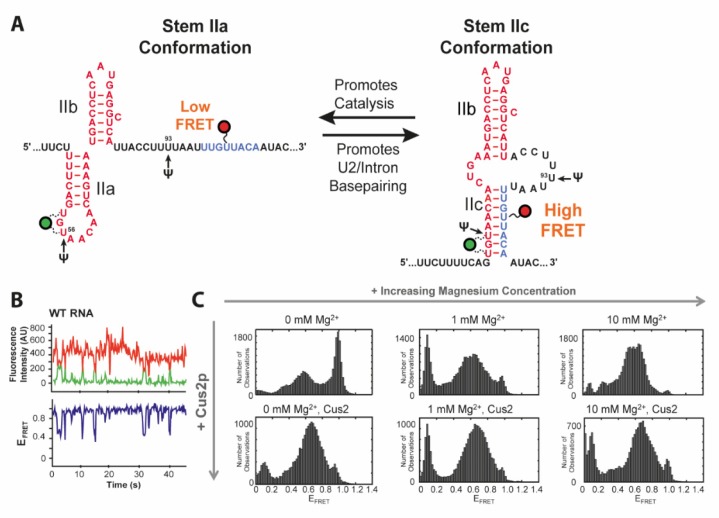
FRET construct and data for toggling of U2 snRNA. (**A**) The stem II of the U2 snRNA can switch between two conformations. Labeling positions in the two studies of Cy3 (green) and Cy5 (red) as well as Pseudouridylation sites are indicated. (**B**) Example FRET trajectory of U2 shows a spontaneous toggling between two FRET states. (**C**) FRET histograms of WT RNA in dependence of Mg^2+^ and Cus2 concentrations. This Figure was adapted and modified from Rodgers et al. [16] and used under Creative Commons Non-Commercial License (CC-BY NC).

The cold-sensitive U2 snRNA suppressor protein 2 (Cus2) assists Prp5 in prespliceosome activation and confers ATP dependency of the remodeling process [35]. It has been proposed that hydrolysis of ATP is required for the subsequent removal of Cus2 [32,35,36]. Throughout the splicing cycle, the U2 snRNA undergoes several conformational changes between the two transesterifications and rearrangements of the pre-mRNA substrate. This primarily affects the stem II region, which can adopt two conformations via folding of either hairpin IIa or IIc (Figure 2A) [37,38]. While IIc is responsible for both catalytic reactions, IIa is necessary for substrate binding and release [37,38]. The latter conformation is presumably bound and stabilized by Cus2 [39]. Splicing efficiency is also dependent on posttranscriptional modifications of U2, such as three conserved pseudouridines [40,41,42] and under distinct stress conditions additionally at positions 93 and 56 [43,44].

Rodgers et al. and van der Feltz et al. addressed this toggling of U2 between stems IIa and IIc using smFRET spectroscopy [16,17]. Discrimination between the two conformations was facilitated by the two states showing either a low FRET (stem IIa) or a high FRET (stem IIc) state (Figure 2C) as designed based on structural data. In the absence of Mg^2+^ analysis of their RNA construct showed coexistence of a high FRET state of 0.96 corresponding to IIc, a FRET efficiency of 0.54 for IIa and an additional, minor low FRET conformation of 0.35, all three dynamically interconverting into each other (Figure 2B,C). smFRET showed the titratable impact of Mg^2+^ or Cus2 as they both individually shifted the equilibrium in favor of the IIa conformation. Wild type transitions to the low FRET state vanished in the presence of Cus2. smFRET could not only reveal the influence of the environment on the ratio between subpopulations and their transition frequency. It can also be applied to evaluate the effects of nucleotide modifications as presented by van der Feltz et al. through introduction of pseudouridine at the stress inducible positions Ψ56 and Ψ93. With pseudouridylation at both positions for instance, neither Mg^2+^ nor Cus2 impacted stem II conformation. Only a combination of Mg^2+^ with Cus2 was able to overcome this stabilization of the IIc conformation caused by the dual modification Ψ56 and Ψ93.

## 5. Observation of the Full Splicing Cycle

The first three studies discussed in this review characterized the dynamic behavior of isolated steps of the splicing process. Throughout the splicing cycle, the spliceosome shows a drastic level of compositional as well as conformational complexity. A thorough description of at least aspects of the splicing cycle therefore requires tools for investigating the system in toto. The heterogeneous occupancy of states, the ability to study unsynchronized processes and their dynamics in real time render a single molecule approach the method of choice for this type of spliceosome analysis.

**Figure 3:**
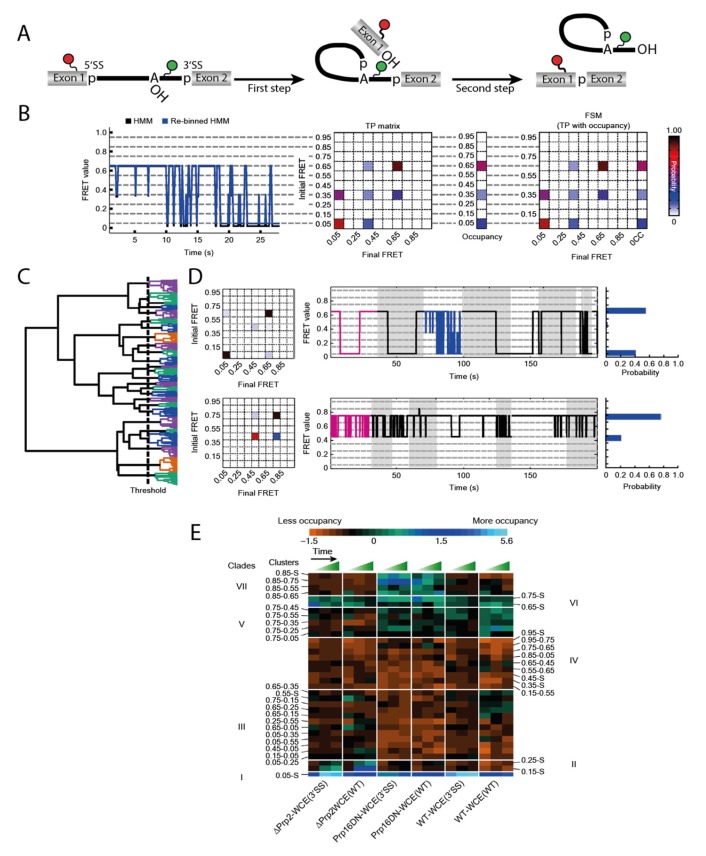
Workflow of the Single Molecule Cluster Analysis (SiMCAn) approach on the full splicing cycle [15]. (**A**) pre-mRNA FRET construct used in this study. During the first splicing step a lariat structure is formed and after splicing the exons are ligated and the intron-lariat released. (**B**) Hidden Markov modeling (HMM) fitted exemplary trace (black) and re-binned trace (blue) with the resulting transition probability (TP) matrix and the FRET similarity matrix (FSM). (**C**) Clustering of the molecules resulting in a hierarchical tree with every leaf representing a single molecule and sorting into 25 clusters indicated by the same color. (**D**) Representation of 2 clusters with their TP matrix, exemplary traces with the best trace in magenta and the occupancy in each state. (**E**) Second round of clustering of the clusters into 7 clades which show similar behavior under changing conditions. © 2015 Nature America, Inc. Used with permission.

Blanco et al. described an algorithm to tackle this problem [15]. Their method Single Molecule Cluster Analysis (SiMCAn) is based on ‘hierarchical clustering of Hidden Markov modeling-fitted smFRET trajectories’ [15]. Each individual FRET trace is first fitted via Hidden Markov modeling (HMM) [45] and subsequently re-binned to one of ten evenly spaced FRET states from 0.05 to 0.95 (Figure 3B). Using the HMM traces, a transition probability (TP) matrix is generated for each molecule which is then additionally described with the occupancy to a FRET similarity matrix (FSM) (Figure 3B). After separating out the static molecules, the dynamic FSMs allow clustering of several thousand molecules according to their FRET behavior, their state occupancy, and transition kinetics. This clustering results in a hierarchical tree sorting the molecules according to similarity in their dynamic behavior (Figure 3C). Each individual cluster contains an average TP matrix, a collection of traces, and an occupancy probability distribution (Figure 3D). While this technique can already identify dynamics that cannot be found by manual sorting, it is necessary to connect the numerous clusters to their function in order to understand a mechanistically complex system such as the spliceosome. In order to obtain data that is sufficient to provide such an understanding, it is necessary to trap the process under investigation at predefined stages to accumulate certain intermediates. Those roadblocks could come in the form of deletions or mutations of components, or the use of specific inhibitors. By generating such intermediates, additional data on the existing clusters can be obtained. In a second round of clustering, the static and dynamic clusters combined are summarized in clades if they show similar enrichment or depletion under the same condition (Figure 3E). This allows for the assignment of the FRET dynamics to the individual steps of the entire system by means of their clades.

The spliceosome substrate, the pre-mRNA, is present at all stages of the splicing cycle and undergoes many conformational as well as chemical changes through the process. A construct that had previously been used for smFRET analysis of the first splicing step dynamics [29] was a pre-mRNA that was labeled at Exon 1 near the 5′SS and near the branchpoint, expecting high FRET when those sites come in close proximity (Figure 3A). The same construct was now been used with a nuclear extract conferring splicing activity in smFRET analysis. While these data are too complex for a manual evaluation because molecules occupy different steps of the splicing cycle, the authors emphasize the advantages of SiMCAn and the amount of information this method provides. When analyzing the highly dynamic and complex data obtained from these experiments, the SiMCAn algorithm found ten static and 25 dynamic clusters, which are further clustered to seven distinct clades [15].

Applying different roadblocks throughout the splicing cycle and observing the (time-dependent) accumulation, the authors could characterize the clade deriving from the commitment complex 2, the A-complex, the B^act^ complex and the post first step C-complex. SiMCAn uncovered that the splicing cycle is not strictly directed forwards. A reversible switching back to the commitment complex 2 and A-complex was identified through dynamic clades. In addition to resolving all the biochemically described steps mentioned above, the SiMCAn technique provided evidence for the existence of a novel proofreading and discarding mechanism for incorrect 3′SS sequences after the first reaction. smFRET in combination with SiMCAn is a powerful tool to analyze multistep reactions as shown by the spliceosome. This technique was not only able to characterize distance changes throughout the full cycle, but also delivered kinetic data as every cluster also contains information about transition rates. Furthermore, new reaction steps were identified in the case of the spliceosome.

In summary, the studies presented here demonstrated how conformational analysis of parts of the spliceosome afford a functional connection between individual components of this highly complex RNP. The level of complexity requires both of the approaches presented here: bottom-up, where individual (sub-) domains of RNAs and proteins are put into focus, and top-down, where a less well-defined splicing activity conferred by cellular extracts are analyzed to yield insights from both directions.

## 6. G-Quadruplex Helicase

The key structural element of the complex protecting the telomeres are intricate DNA structures consisting of three guanosine tetrads, termed G-quadruplexes (G4s) [46]. These structures have been described for a large number of nucleic acid species, including not only chromosomal DNA termini, but presumably also in various other functionally relevant genomic regions and RNAs [47,48,49,50]. They are however evolutionarily depleted in bacterial transcriptomes [51].

The structure of these G-quadruplex elements depends on various factors, such as sequence composition, loop size, the cation bound into the quadruplex core, and the number of molecules involved in formation of quadruplex structures, i.e., inter- or intramolecular structures [47,49,52]. In general, all quadruplexes exhibit very high thermal stability, with RNA G4s usually exceeding the stability of DNA G4s. At the same time, RNA G4s are usually in a parallel orientation and show lower structural diversity than DNA G4 structures [53,54].

Aside from their function within telomeres, these structures have been described to be functionally relevant in processes like transcriptional pausing, replication, translation, and RNA processing. They exert their effect by utilizing the intrinsic thermal stability, leading to steric interference, competition with other conformations, or as recruiting platform for other proteins [54].

The broad spectrum of structures G4s are able to adopt, and their broad variety of binding partners as well as their intrinsic dynamics render these structures extremely difficult to study in their functional context. It is therefore not surprising that studies focusing on G4 structures in complex with e.g., protein binding partners are scarce.

There have been a number of proteins described that are able to unfold G4 structures in DNA as well as in RNA [53]. The most prominent member of RNA G4 helicases is the G4 resolvase RHAU (also termed DHX36 or G4 resolvase 1) which was first identified in 1997 [55]. It is able to resolve both inter- and intramolecular G4 in DNA and RNA [56,57,58] and its physiological importance is exemplified by its role in telomere reverse transcription. RHAU unfolds the intramolecular G4 in the 5′-region of human telomerase RNA template (hTR) and thereby promotes formation of the P1 helix, which prevents misincorporation during reverse transcription by serving as physical barrier [59].

Recent research on the RNA G4 helicase RHAU is focusing on the binding mode and unwinding mechanism on intramolecular DNA and RNA G4s. However, structural data obtained from crystal structures and small angle X-ray scattering (SAXS) even in combination was not sufficient to characterize the unwinding process, as it provided only snapshots. The authors therefore opted to complement their structures of different complex compositions (i.e., in absence and presence of cofactors) by employing smFRET to investigate the conformationally dynamic nature of the unwinding process.

**Figure 4:**
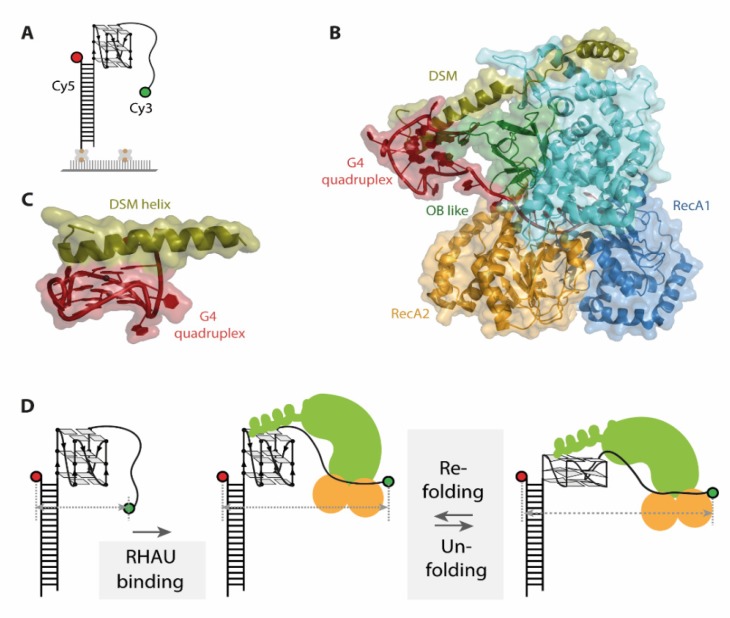
(**A**) Schematic representation of one G-quadruplex (G4) single molecule Förster resonance energy transfer (smFRET) construct used in the described studies. (**B**) Co-crystal structure (pdb 5VHE) of RHAU in complex with a c-Myc G-quadruplex, color-coded for different important protein domains. (**C**) The DSM helix of RHAU stacks as planar, unipolar surface on top of the G-quadruplex. (**D**) Unfolding mechanism of DNA G4s by RHAU derived from smFRET studies. Binding of RHAU to the G4 results in FRET decrease, followed by ATP independent, repetitive partial unfolding and refolding of DNA G4. Figures **A** and **D** were adapted and modified from Tippana et al. and are used under Creative Commons Attribution 4.0 International License (CC-BY 4.0).

Previous structural and biochemical data revealed selective binding of RHAU to parallel DNA as well as RNA G4 [52,60]. smFRET data on structurally diverse G4s supported this finding, identifying RHAU binding only to the fraction of G4s corresponding to a parallel G4 fold [19]. For this study, an approach using immobilized substrate G4s was applied. To assess the binding and unwinding of the G4, donor and acceptor fluorophore were attached at opposite ends of the nucleotide strand (Figure 4A). A completely folded G4 brings the two fluorophores in close proximity and therefore exhibits high FRET efficiency values. Binding and unfolding of the G4 structure leads to a decrease in FRET efficiency, resulting in lower FRET efficiency values for an unfolded state. Several later studies used analogous labeling schemes to assess the unwinding mechanism of RHAU on G4s as they capture conformational changes in G4s along the whole process [20,21,22].

Moreover, a single stranded overhang of at least 9 nucleotides is required for efficient RHAU binding to G4s [19,57]. Later studies on bovine RHAU however indicate the possibility of recognizing antiparallel G4s as well [20,60], and binding of G4s regardless of their structure was shown for RHAU from *Drosophila*. In general, there are a number of differences described between bovine and Drosophila RHAU, showing why a detailed analysis at the level of individual molecules is of advantage.

The co-crystal structure of the DEAH-box helicase RHAU and a G4 DNA individually describes the importance of several protein domains (RecA1 and 2, DSM and OB like, see Figure 4B) for G4 binding as well as unwinding [20,22]. The G4 core is located in a positively charged cage, stacked under a DSM domain helix (Figure 4C). This structural element is essential for substrate binding in bovine, but not in drosophila RHAU [20,22,60]. smFRET assays analyzing this binding mode in detail used immobilized DNA and RNA G4s together with several mutants targeting the DSM helix of bovine RHAU. Dissociation of RHAU from DNA G4s upon buffer flow demonstrate the requirement for an intact DSM helix for tight DNA binding. However, in the same experimental setup, no dissociation from RNA G4s was detected, hinting towards a different role of DSM in RNA G4 binding [20,21].

The conformational dynamics of the G4 unwinding mechanism by RHAU were then investigated via smFRET. In fact, different unwinding pathways were reported for DNA and RNA G4s [20,21,22]. For DNA, binding of the helicase to the substrate G4 is followed by repetitive unfolding and refolding of the quadruplex as evidenced by switching between two FRET states at 0.35 and 0.6, respectively [19] (Figure 4D). The observed FRET efficiency oscillations were elegantly shown to arise from a single RHAU molecule rather than protein dissociation and rebinding, as immobilizing the protein instead of the substrate did not alter the observed FRET behavior. An additional dwell time analysis of FRET states uncovered that the unfolding and refolding time of this process is closely related to the thermal stability of the G4, with faster refolding rates for more stable G4s. The repetitive unfolding mechanism would have remained elusive by structural approaches, which only captured the intermediate conformations in this cycle.

Comparison of the unwinding dynamics to structural data impressively augmented the understanding of the unwinding process. The unfolding and refolding represents a repetitive pulling mechanism on the 3′-most G in a direction outwards of the quadruplex. This pulling reorganizes the canonical G4 and results in formation of an ATGG quartet at the 5′ end of the quadruplex, which is stacked on top of two canonical guanosine tetrads. Within the 3′ single stranded region of the G4 this repetitive pulling results in an alternating stacking between 4 and 5 nucleotides in the protein tunnel [20,22].

The abovementioned mechanism is however ATP independent, and only leads to partial unfolding of the G4 structure [20,22]. Nevertheless, Tippana et al. could show by smFRET assays that surprisingly, RHAU-mediated pulling of one single nucleotide from the G4 structure is sufficient to destabilize the quadruplex to such an extent, that a complementary cis-strand can efficiently anneal. This is indicated by a stable low FRET value, matching the measured FRET value of the completely unfolded G4. Moreover, addition of ATP did not change the annealing or unfolding behavior [19]. The role of ATP in this unwinding process was reported to be different for bovine and drosophila RHAU [20,22].

**Figure 5:**
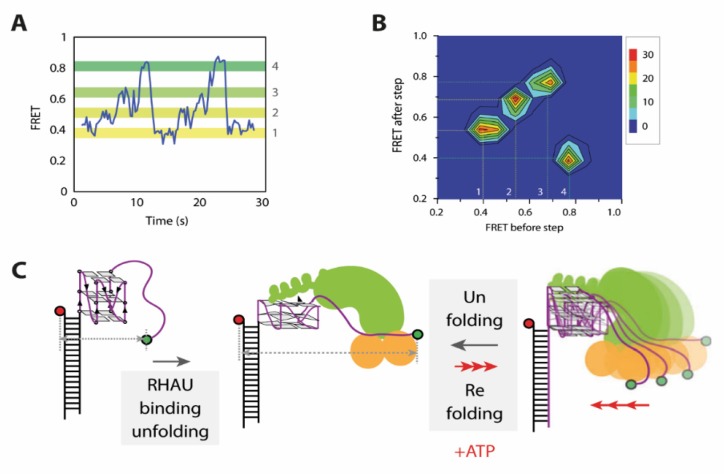
Unfolding mechanism of RNA G4s by RHAU. (**A)** FRET traces of ATP dependent repetitive unfolding and stepwise refolding. (**B**) Transition density plots (TDPs) generated from FRET traces. (**C**) Unwinding mechanism of RNA G4s by RHAU developed by smFRET studies. Initial binding of RHAU induces partial unfolding, followed by ATP dependent, repetitive stepwise refolding and successive unfolding. This Figure was taken from Tippana et al. and used Creative Commons Attribution 4.0 International License (CC-BY 4.0).

Based on smFRET studies, the unwinding mechanism of RNA G4s by RHAU was very recently investigated by Tippana et al., describing a significantly different conformationally dynamic nature than that of DNA G4 unfolding by RHAU [21]. Binding of RHAU to RNA G4s results in a two-step decrease from a high FRET state at 0.8 to an intermediate state at 0.6, and then to a low FRET value of 0.4, which up to this point is similar to the ATP independent partial unfolding of DNA. However, after binding (E_FRET_ 0.6) and partial disruption (E_FRET_ 0.4) of the G4, no FRET fluctuations between the two states were detected, which indicates that there is no refolding step in the absence of ATP. Upon addition of ATP, refolding of the G4 by RHAU was observed in three distinct steps. The last step of refolding was followed by a rapid decrease back to a FRET state at 0.4, which was assigned to be a second single-step unfolding (Figure 5A). Transition density plots (TDPs) of the FRET traces were able to confirm that this is a closed cycle, showing no additional interconversions (Figure 5B). In contrast to the faster DNA G4 repetitive unwinding by RHAU, the significantly slower unfolding and refolding cycle of RNA G4 was observed repeatedly in the presence of ATP, prior to RHAU dissociation from the substrate RNA (Figure 5A,C). Similar to studies on DNA [19], an approach using immobilized RHAU confirmed that this motion arises from a single RHAU molecule and is not due to repeated docking and dissociation. This is a perfect example of how smFRET spectroscopy is able to complement existing structural data with relevant kinetic information and interconversion pathways between intermediates.

The ATP dependence of the RNA G4 refolding step by RHAU arises from the presence of a 3’ single stranded RNA overhang [21]. Innovative smFRET constructs built from RNA-DNA chimeras (DNA G4–RNA overhang, RNA G4–DNA overhang) showed significant differences in RHAU-mediated unfolding dynamics. Constructs with a DNA overhang and an RNA G4 exhibited the FRET fluctuations known from DNA G4 unfolding by RHAU, whereas constructs with an RNA overhang displayed movements similar to RNA unfolding dynamics. The behavior upon ATP addition is also analogous, showing an ATP independence for unwinding of the RNA G4–DNA overhang chimera, and a strict ATP dependence for unwinding of the DNA G4–RNA overhang chimera.

The de facto unwinding of the RNA G4 by RHAU was proven by cis-strand annealing to the RNA. Under ATP hydrolysis, efficient annealing was observed, indicated by a stable low FRET state that was persisting under SDS-buffer flow. Without addition of ATP or with ATPase deficient RHAU mutants, SDS-buffer flow induced a refolding of the G4 due to dissociation of RHAU from the substrate, shifting the FRET values back to high FRET.

When looking at the studies on G4 unwinding presented here, smFRET based findings were able to complement the structural data to a remarkably clearer understanding of the conformationally dynamic nature of the unwinding process, experimentally linking individual structures to a distinct mechanism: First, the conformational dynamics of the G4 structures throughout the unwinding process show that the interactions between the protein and the G4 are more complex than anticipated and differences kinetics between RNA G4s and DNA G4s The differences in ATP-dependence was also surprising, and the analysis using RNA-DNA chimera constructs elegantly isolated the individual contribution of structural elements on the unwinding dynamics. All of these findings nicely complement the information on the same complexes available from structural analysis.

## 7. Telomere Maintenance

**Figure 6:**
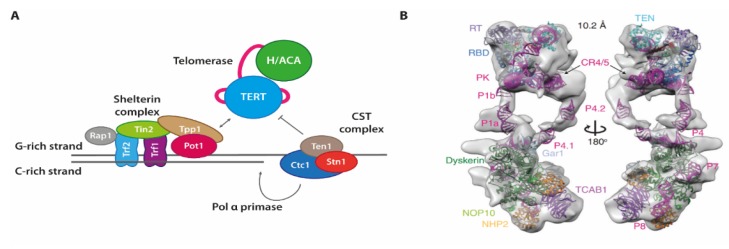
(**A**) Protein and RNP complexes at the chromosome ends. Whereas the shelterin complex binds to protect the chromosome ends, telomerase as well as the CST complex act on the G-rich or C-rich strand, respectively, to facilitate their synthesis. (**B**) Cryo-EM-based model structure of the human telomerase holoenzyme, taken from [63]. Copyright of Figure 6B by Wang et al. is with CSHP—Copyright for use has been requested from the publisher.

Each of the chromosomes in eukaryotic cells consists of linear double-stranded DNA (ds DNA), which also contains termini of the linear DNA at each end. In order to prevent undesired recombination or other erroneous processing of these DNA ends (as they may mistakenly be recognized as double-strand breaks), complexes termed shelterin are placed onto and shield each of the chromosome ends [61]. These proteins also help regulate the selective elongation of both terminal DNA strands, and are thus a key player in maintaining a specific length of the telomeres [62] (Figure 6). To selectively evade the shortening of telomeric sequences during cell division, cells use a specialized system consisting of two complexes each synthesizing one strand of the genomic DNA. For the G-rich strand that is synthesized de novo, an RNP containing a reverse transcriptase with an integral RNA template form the telomerase enzyme. For the C-rich strand, the G-rich strand is used as a template. Here, the CST-complex consisting of three proteins Ctc1, Stn1 and Ten1, recruits the Pol a-Primase, and also inhibits telomerase.

Telomerase holoenzyme RNP complexes are assembled and processed in the nucleus and nucleolus before being selectively localized at chromosome ends [64]. The use of an integral RNA template to add repetitive DNA sequences requires that a short duplex of RNA template and the newly synthesized DNA are shifted for each nucleotide added to allow for consecutive addition of bases (“nucleotide addition processivity”). After reaching the end of the RNA template, the duplex has to be realigned to allow for the synthesis of a new repeat (“repeat addition processivity”).

The unique architecture and functionality of telomerase enzymes has made a detailed functional characterization as well as structural biology approaches difficult for a long time. Initial, partial structures of protein domains from diverse organisms including the reverse transcriptase TERT [65,66], accessory proteins like the Tetrahymena p65 [67], or parts of RNA elements within the RNA component [68,69] have provided some insight. Fully assembled complexes however were extremely difficult to obtain [70]. This was changed by more recent cryogenic electron microscopy (cryoEM) structures first on the Tetrahymena holoenzyme [71], and later on high-resolution structures of Tetrahymena telomerase [72] as well as the human telomerase holoenzyme [73].

Together with a number of single molecule-based approaches, the structures also shed light on aspects of telomerase subunit folding [74,75,76,77], as well as on the general conformational dynamics that are required for telomerase activity [78,79].

**Figure 7:**
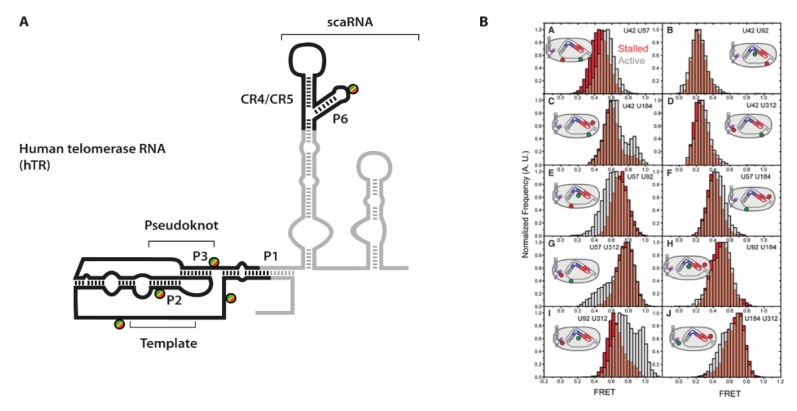
(**A**) Secondary structure of human telomerase RNA hTR with labeling sites. The parts in grey are representing the full RNA, while the parts in black have been used in the study [24]. (**B**) smFRET histograms derived from the ten labeling site combinations. Data derived from the stalled complex is depicted in red, while complexes selected for activity are in grey. Figure 7B is taken from Parks et al. and used under Creative Commons Non-Commercial License (CC-BY NC).

Especially for the human telomerase holoenzyme however, the overall arrangement of the RNA component relative to TERT was difficult to establish. One particular obstacle was the discontinuous set of structural and functional elements within hTR (see Figure 7).

In order to elucidate the orientation of these domains towards each other as well as upon binding to TERT, Parks et al. combined an extensive set of smFRET experiments with a modeling approach [24]. They placed FRET pairs on five positions into different parts of hTR, reconstituted these RNAs with TERT into functional constructs and derived distance constraints from these experiments. The distances were strategically placed into each of the hTR domains: close to the P1 helix, two positions on either side adjacent to the pseudoknot, 3′ of the template sequence, and within the CR4/CR5 element P6 helix.

To efficiently immobilize fully reconstituted telomerase holoenzyme species, a specific primer substrate showing high affinity towards the assembled complex was used [80]. In absence of dNTP substrates, these complexes are referred to as “stalled” complexes. In order to then obtain data throughout the telomere synthesis cycle, the “active” complexes were imaged after addition of substrate dNTPs [81]. Conformational dynamics that occur within hTR would then result in a different distance to be reported in FRET experiments. Indeed, for several of the ten labeling combinations, differences between “stalled” and “active” complexes were observed. The conformational shifts expectedly were not quantitative (see e.g., Figure 7), as several studies showed that a large fraction of human telomerase complexes that can be isolated from cells are actually catalytically inactive [64,73]. However, the additional FRET populations allowed deriving of an additional set of distances within the telomerase holoenzyme. Interestingly, the differences were mostly observed in the orientation of either the template sequence and the pseudoknot structure, or between the pseudoknot structure and the CR4/CR5 hairpin, respectively.

**Figure 8:**
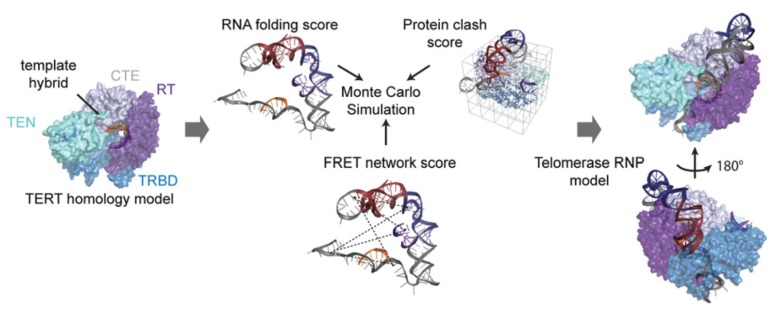
Workflow to derive structural models of higher quality by incorporating the FRET network score into Rosetta modeling. With existing structural data from homology models, the authors combined restraints obtained from RNA folding, steric calculations and smFRET data into a novel model of the hTR-TERT RNP. This Figure is taken from Parks et al. and used under Creative Commons Non-Commercial License (CC-BY NC).

The additional information derived in this study was however obtained by including the distance restraints derived from FRET experiments into a structural model of hTR architecture that was in line with previous experimental datasets [62,79]. Considering functional restraints, a clustered Rosetta modeling approach was used to obtain a likely fold for hTR in complex with TERT. The authors showed that only when considering the FRET restraints, these models were convergent (Figure 8). These models were in line with the models available for the Tetrahymena telomerase at that time. Rewardingly, these models were also in excellent agreement with the structural data of human telomerase generated by cryoEM later on [73]. Even considering dynamic movements within the holoenzyme, the study produced viable models for conformations that can be assumed throughout the active cycle.

While telomerase provides one factor to extend the successively shortened G-rich strand, the C-rich complementary strand also has to be selectively synthesized (Figure 6). In order to exert control over both quadruplex formation and telomerase, human cells employ the CST complex, consisting of proteins Ctc1, Stn1, and Ten1. This complex limits the activity of telomerase [82], unwinds the G-rich DNA quadruplex structures [83] to allow for the “fill-in” reaction of the C-rich strand by Polα, and by combination of these processes limits the length of the overhang formed by the G-rich strand [84].

**Figure 9:**
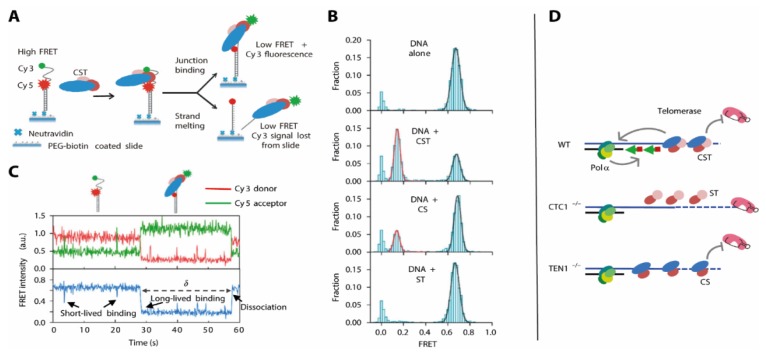
(**A)** smFRET construct design to investigate G4 resolvase activity of the CST complexes. (**B**) smFRET data depicting the efficient unfolding of the G4 construct by CST, less efficient unfolding in presence of CS, and no unfolding with ST. (**C**) Time-resolved unfolding and identification of intermediates in presence of CST. (**D**) Model for the interplay between the CST complex and Polα. This Figure is adapted from Feng et al. [23] and used under Creative Commons Non-Commercial License (CC-BY).

How the CST complex is able to make the G-rich strand accessible for further DNA synthesis is less well understood. Here, an approach combining biochemical and smFRET analysis of DNA duplexes with different overhang lengths and positions in presence of the CST complex provided deeper insight [25]: In electrophoretic mobility shift assay experiments, high affinity of CST to telomeric overhang structures shows that the site where the dsDNA transitions into single stranded (ss) DNA at the G-rich strand is specifically recognized and destabilized by CST. As a biochemical assessment of these heterogeneous systems was difficult, the authors used an smFRET approach where – similar to other G4 enzymes (Figure 5)–a folded DNA was immobilized and G4 stability monitored by FRET. Analysis of these biochemically identified structures unveiled that CST is also able to unwind the G4 structure in the context of the ds-to-ss-DNA junction (Figure 9A). This was also the case when there was a non-telomeric overhang present, showing that the unfolding was structure- rather than sequence-specific.

Binding of CST in situ caused an immediate shift into a low-FRET, G4 unfolded conformation (Figure 9B), and a number of additional experiments where the protein trimer was added during the experiment showed that this process was fully reversible (Figure 9C). This finding immediately puts forward a model where the specific recognition of the overhang junction and the immediate destabilization of the G4 structure facilitate the elongation of the C-rich strand, using the unstructured G-rich strand as a template.

These findings however were derived from the CST trimer in absence of in-depth functional knowledge about any of the proteins. These individual contributions were then addressed in a follow-up study [23], showing that for Ctc1-depleted cells, there is both an increase in length for the G-rich strand and a length decrease in the C-rich strand.

To test how these different observations were exerted by the different subunits of the CST complex, the authors expressed and purified “CS” and “ST” complexes containing Ctc1-Stn1 and Stn1-Ten1, respectively. They assessed the G4 unfolding activity of these complexes in smFRET experiments and compared them to the results using the full CST complex (Figure 9). They found that the ST complex was unable to resolve the G4 structure within the G-rich strand, indicated by an unchanged high-FRET population. The CS complex had activity that was reduced, which was indicated by a difference in short-lived binding events between the DNA G4 and the CS complex. The resulting unfolded state of the DNA however was comparable to that generated by the CST complex. This is in perfect agreement with the results using cell line analysis: While CST both recruits Polα and inhibits telomerase activity, these functions can be separated for the subcomplexes (Figure 9D). Here, the ST complex (in absence of Ctc1) allows for G-rich strand elongation by losing its telomerase inhibitor function. The CS complex in absence of Ten1 however diminishes telomerase activity, but lacks the feature to recruit Polα in order to fill in the C-rich strand.

This again provides an elegant example how carefully crafted smFRET experiments are able to fill in gaps in the functional understanding of such processes by comparative analysis of subcomplexes, and the combination of such experiments with biochemical assessment of substrate recognition.

## 8. Conclusions

Taken together, the studies on various RNP complexes presented here all clearly show that strategic smFRET experiments can significantly improve the understanding of complex systems, on their conformationally dynamic nature but just as much on their structural composition.

The research on the spliceosome summarized here shows how smFRET was able to shed light onto the complex rearrangements necessary throughout the splicing cycle, where structure determination such as crystallization and cryo-EM only delivered snapshots. With different labeling schemes on either protein, snRNA, or substrate RNA it was possible to detect the RNA- and ATP-dependent closing of Prp5, the spontaneous or assisted conformational toggling of U2 snRNA in dependence of ions, proteins and modification as well as pre-mRNA dynamics throughout the whole splicing cycle. While some of these principles have been hypothesized previously, single-molecule data could provide crucial evidence for their existence. Furthermore, these smFRET studies were able to additionally find and visualize novel intermediates and pathways.

The presented studies on G4 unwinding by RHAU show the benefit of single molecule experiments when combining them with structural data on RHAU. The identification of distinct states throughout the unwinding process, the dynamics that come along with both the interaction with the enzyme and the ATP hydrolysis step and, finally, the impact of mutations on such dynamics all would have been extremely difficult to rationalize from structural or biochemical data alone.

The unique telomerase mechanism proved to be especially challenging to decipher. The smFRET data gave significantly deeper insights into many of the aspects in telomerase folding, assembly and activity, and demonstrated how questions that were again difficult to tackle by structural biology means were more readily amenable by single molecule techniques. Telomerase holoenzyme structures published in the last years confirmed that several previously hypothesized insights into the mechanism of telomerase-catalyzed DNA synthesis were correct. This mostly concerned the binding locations of proteins and their interactions with the RNA, but also the general architecture and arrangement of the RNA within the telomerase holoenzyme. They also further structurally explained how the telomerase cycles during DNA synthesis, combining nucleotide addition processivity and repeat addition processivity to efficiently add significant amounts of DNA onto the chromosome ends.

By looking at strategically reconstituted subcomplexes, it was again the view into individual molecules that precisely identified functional roles for each protein within the CST complex.

In summary, the work showcased here provides a number of prominent examples in how functional questions on RNP complexes can be tackled by combining and comparing structural biology techniques with FRET spectroscopy on the level of individual molecules.
